# Paclitaxel Induce Apoptosis of Giant Cells Tumor of Bone *via* TP53INP1 Signaling

**DOI:** 10.1111/os.12414

**Published:** 2018-12-27

**Authors:** Wei‐Yuan Xiao, Zhen Zong, Man‐Le Qiu, Xiu‐Yuan Chen, Hong‐Xing Shen, Li‐Feng Lao

**Affiliations:** ^1^ Department of Spine Surgery, Renji Hospital, School of Medicine Shanghai Jiao Tong University Shanghai China

**Keywords:** Apoptosis, giant cell tumors of bone, paclitaxel, TP53INP1 signaling, transcriptomics

## Abstract

**Objective:**

To evaluate the antitumor capability and to investigate the underlying molecular mechanism of paclitaxel.

**Methods:**

First, cck‐8 and apoptosis assays were used to determine survival and apoptotic effects of HS 737.T cells under treatment of paclitaxel. Next, RNA‐seq and bioinformatics were used to determine the differentially expressed genes and to analyze the pathway involved. Quantitative real‐time polymerase chain reaction was used to verify the accuracy of some differentially expressed genes (DEG). ClueGO was used to decode and visualize functionally grouped GO terms of differentially expressed genes, and to map the DEG protein–protein interactions (PPI) network. Western blotting was used to check the expression of target genes, the cleavage of Caspase‐3 and PARP1, and the phosphorylation level of p53. Finally, transcriptomics, bioinformatics, and RNAi were used to estimate the antitumor capability and to identify the underlying mechanisms of paclitaxel in GCTB.

**Results:**

Our data revealed that paclitaxel had significant time‐dependent effects on the viability and induced apoptosis of HS 737.T cells. RNA‐seq and bioinformatics analysis showed that apoptosis, death receptor signaling pathway, TNF signaling pathway, and TP53 regulated transcription of cell death genes pathway were closely associated with paclitaxel in the treatment of GCTB. Western bolt results revealed that paclitaxel induced cleavage of Caspase‐3 and PARP1, and increased the phosphorylation level of p53 in HS 737.T cells. RNAi results showed that the expression level of TP53INP1 was significantly decreased in HS737.T cells (the decrease was more than 70%). In addition, we found that the inhibitory ratios of paclitaxel on HS737.T cells deficient in TP53INP1 were less than in HS737.T cells with empty vector (19.88 and 40.60%, respectively). Hence, our data revealed that TP53INP1 regulated paclitaxel‐driven apoptosis in HS737.T cells.

**Conclusion:**

Paclitaxel can significantly repress cell proliferation and induce apoptosis of HS 737.T cells through activating Caspase‐3, PARP1, p53, and TP53INP1. Paclitaxel may be an effective drug in the management of GCTB.

## Introduction

A giant cell tumor of bone (GCTB) is an osteolytic, locally aggressive, rarely metastasizing bone tumor. Many GCTB are benign connective tissue neoplasms, such as stromal cells, osteoclast‐like giant cells, and tumor‐associated monocytes/macrophages^1^. The stromal cells represent the neoplastic component of the tumor, because of their capacity of proliferation and invasion to peripheral tissue^2^.

It is well known that the transformation of cancer cells leads to an increasing trend of apoptosis. Therefore, apoptosis of multinucleated giant cells and stromal cells may increase in GCTB to regulate tumor regression^3^. Our understanding of the anti‐proliferation and apoptosis mechanisms will enable us to put forward more rational methods of cancer treatment.

Paclitaxel, which is derived from the bark of the Pacific yew tree, *Taxus brevifolia*, exhibits potent efficacy against both breast and ovarian cancers. Mechanistically, paclitaxel selectively binds to tubulin and subsequently stabilizes microtubules, thus inhibiting cell division. Interestingly, metastatic bone lesions have been reported to better respond to a combination of paclitaxel and docetaxel than to a variety of single chemotherapeutic agents. While these clinical observations point to a beneficial effect of paclitaxel on bone lesions, the underlying mechanisms of its action remain to be established. Numerous studies have demonstrated that paclitaxel has a significant role in the modulation of the cell cycle and apoptosis in various tumor cells. Nevertheless, the mechanisms by which paclitaxel modulates the anti‐proliferation and apoptosis of GCTB remain obscure.

Apoptosis plays a crucial role in anticancer and disease defense. In general, the caspase‐dependent apoptosis is driven by interior or exterior stimuli^4^. Exterior pathways are involved in the death receptor ligands (like TRAIL and TNF), which bind to their receptors (such as TNF‐R1, DR3, DR4, and DR5)^5^, ^6^, ^7^. When the ligands bind to death receptors, their cytoplasmic domains recruit adaptor molecules and trigger caspases’ cascade. Finally, they activate Caspase‐8 and Caspase‐10, which subsequently results in the activation of effector caspases, such as Caspase‐3 or Caspase‐9^7^, ^8^. The interior pathway of apoptosis destroys the mitochondrial membrane and releases apoptosis‐associated proteins, like cytochrome c, which successively activates Caspase‐9 and Caspase‐3, inducing apoptosis^9^, ^10^.

In the present study, to further elucidate the anti‐tumor activity and mechanisms of paclitaxel on GCTB, we used RNA‐Seq, RT‐qPCR, and western blot, to reveal differentially expressed genes in the giant cell tumor cell HS 737.T under paclitaxel treatment. We then used bioinformatics to analyze the pathway that the differentially expressed genes involved. Finally, we combined transcriptomics, bioinformatics, and RNAi to investigate the underlying mechanisms of paclitaxel on GCTB.

## Materials and Methods

### *Materials*

Paclitaxel was obtained from Selleck (Houston, TX, USA). Cell culture products were from Biological Industries (Cromwell, CT, USA). Puromycin, streptomycin and penicillin, *Lipofectamine* 3000 Transfection Reagent, RT reagent Kit, and quantitative polymerase chain reaction (qPCR) kits were from Thermo Fisher Scientific (Waltham, MA, USA). The CCK‐8 kit was provided by Dojindo Molecular Technologies (Kumamoto, Japan). Restriction enzymes EcoRI‐HF and AgeI‐HF were provided by New England Biolabs (Ipswich, MA, USA). pLKO.1‐TRC cloning vector was a gift from David Root (Addgene plasmid # 10878; http://n2t.net/addgene:10878; RRID: Addgene_10878).

### *Cell Line and Cell Culture*

Human GCTB Hs 737.T (ATCC, USA) were grown in Dulbecco's modified eagle medium plus 10% FBS, l00 μg/mL streptomycin and penicillin.

### *Cell Viability Assay*

Cell viability was checked with a CCK‐8 kit. Hs 737.T cells were exposed to paclitaxel at different concentrations (0–20 ng/mL). After 6–48 h incubation, 20 μL CCK‐8 agent was added to each well; after incubation for 1.5 h, signals were measured using a Microplate Absorbance Reader (Bio‐Rad Laboratories, USA).

### *Flow Cytometry*

To investigate the impact of paclitaxel on apoptosis of GCTB, Hs 737.T cells were treated with or without paclitaxel for 12 h. Next, adherent cells were trypsinized and stained with an Annexin V‐FITC Apoptosis Staining/Detection Kit (Abcam, Cambridge, MA, USA) following the manufacturer's instructions. The apoptotic cells were quantified by flow cytometer (Thermo Fisher Scientific, USA).

### *RNA‐seq Analysis*

RNA‐Library preparation and sequencing were conducted by Novogene (Beijing, China). RNA libraries were constructed with the Illumina mRNA sample preparation kit (Illumina, San Diego, CA, USA) following the manufacturer's directions. Sequencing was performed using the Illumina NextSeq500 platform (Illumina, San Diego, CA, USA).

### *Bioinformatics Analysis*

We used the DESeq to perform differentially expressed gene (DEG) analysis (*P*‐value < 0.05, fold change > 2). Systematic and integrative analysis of DEG was conducted using DAVID bioinformatics resources as previously described^11^, ^12^. Pathway analysis and GO Analysis were applied to determine the roles the DEG played in these biological pathways or GO terms. We also used ClueGO (Cordeliers Research Center, Paris, France) to decode and visualize functionally grouped GO terms of DEG, and to map the DEG protein–protein interactions (PPI) network, following the protocol of Bindea *et al*.^13^.

### *Preparation of RNA and Quantitative Real‐time Polymerase Chain Reaction*

Total RNA was isolated with TRIzol Reagent. RNA integrity, concentration and purity were measured. Reverse transcription PCR was performed using the RT Reagent Kit (Thermo Fisher Scientific, Waltham, MA, USA).

The mRNA levels of interesting genes were checked by quantitative real‐time polymerase chain reaction (RT‐qPCR) using the SYBR Green Real‐Time PCR Kit (Thermo Fisher Scientific, Waltham, MA, USA). The qPCR primers specific for the target genes are presented in Table S1. The qPCR was performed as follows: 95°C for 25 s, 59°C for 25 s, and 72°C for 25 s, 40 cycles.

### *Western Blot Analysis*

Total or intracellular protein were extracted from Hs 737.T cells treated and untreated with paclitaxel, and separated by SDS‐PAGE, then transferred to PVDF membrane. The specific antibodies of the immunoblotting were as follows: anti‐β‐actin, anti‐PARP1 (Cell Signaling, Danvers, MA), anti‐cleaved Caspase‐3, anti‐DDIT4, anti‐TRAIL, anti‐phospho‐p53 (S15), and anti‐TP53INP1 (Abcam, Cambridge, MA, USA). The immunoblot signals were measured and quantified using the Chemiluminescence image analysis system (Tanon Science & Technology, China).

### *RNAi*

To generate Hs 737.T cells deficient in TP53INP1, we used the protocol published previously^14^. Briefly, the shRNA recombined plasmid was generated by ligation of TP53INP1‐targeting shRNA fragment and the pLKO.1‐TRC plasmid (double digested with restriction enzymes EcoRI‐HF and AgeI‐HF). shRNA primers for TP53INP1 can be found in Table S2. The recombinant plasmids were verified by DNA sequencing. pLKO.1 plasmid encoding TP53INP1‐targeting shRNA was transfected into 293T cells by *Lipofectamine* 3000 Transfection Reagent (Thermo Fisher Scientific, Waltham, MA, USA). Lentiviral particles were generated and infected into HS 737.T cells. Stable integration of lentivirus was achieved through selecting culture‐media plus 2 mg/mL puromycin for 48 h. The knockdown efficiency of TP53INP1 was verified by RT‐qPCR or western blot analysis.

### *Statistical Analysis*

Statistics were obtained using Graphpad prism 7(GraphPad Software, San Diego, California, USA). *t*‐test, ANOVA, and Dunnett's and Tukey's multiple comparison test were used to evaluate the statistical significance of the differences. Results were presented as means ± s.e.m. of triplicate experiments. Results were considered statistically significant with *P*‐values < 0.05, *P*‐values < 0.01, and *P*‐values < 0.001.

## Results

### *Inhibited Proliferation and Survival of HS 737.T Cells*

**Figure 1:**
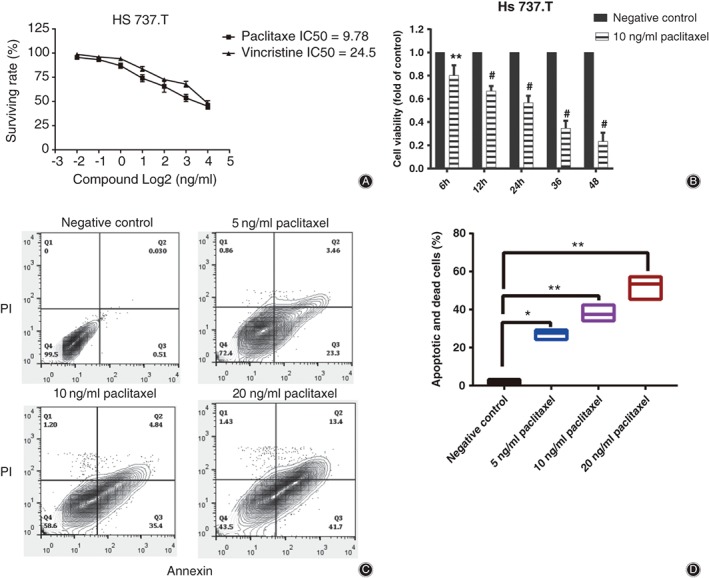
Impact of paclitaxel on the viability and apoptosis of giant cell tumor of bone. (A, B) Effect of paclitaxel on the viability of HS 737.T cells. (C, D) Effect of paclitaxel on the apoptosis of HS 737.T cells; all samples were espoused to various paclitaxel concentrations (0–20 ng/mL) for 12 h. (C) FITC Annexin /PI staining. (D) Each value represented the mean ± SD of the data from three experiments. *P < 0.05, **P < 0.01, #*P* < 0.01 indicated statistical significance versus negative control.

Cell viability experiments demonstrated that paclitaxel was effective in inhibiting cell proliferation and survival of HS 737.T. The IC50 value of the paclitaxel‐treated HS 737.T cell was 9.78 ng/mL (Fig. 1a). At the same time, our data exhibited that paclitaxel had significant time‐dependent effects on the viabilities of HS 737.T cells at 6, 12, 24, 36, and 48 h, with inhibitory ratios of 19.83%, 33.23%, 43.33%, 65.33%, and 76.60%, respectively (Fig. 1b).

### *Enhance Apoptosis of HS 737.T Cells*

Compared with the untreated cells, apoptotic cells were obviously increased after HS 737.T cells were exposed to paclitaxel at 5, 10, and 20 ng/mL for 24 h, with apoptotic ratios of 27.03%, 37.90%, and 52.16%, respectively (Fig. 1c,d). These results further revealed that paclitaxel induced cell apoptosis of HS 737.T.

### *RNA‐seq and Bioinformatics Analysis*

**Figure 2:**
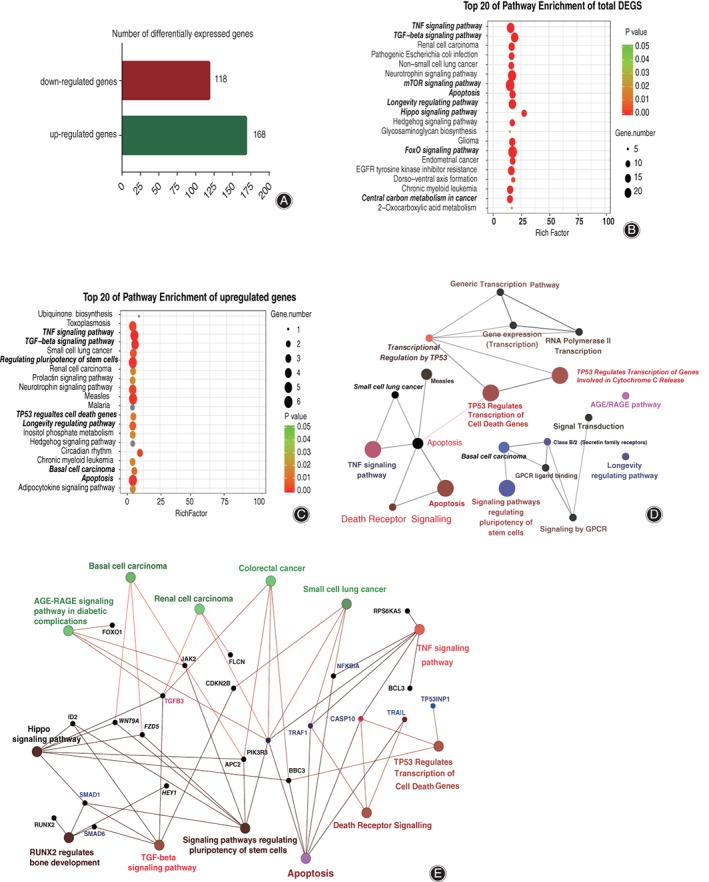
Bioinformatics analysis of differentially expressed genes in HS 737.T cell under paclitaxel treatment compared with negative control. (A) The diagram illustrates the differentially expressed genes among HS 737.T cell samples from RNA‐sequencing results. Functional annotation of the total differentially expressed genes (B) or the upregulated genes (C) between paclitaxel treatment and negative control. Significant changes (*P*‐value <0.05) occurred in the top 20 pathways. Colors represent *P*‐values and dots represent the number of genes involved in each pathway. Functional map of the upregulated gene protein–protein interaction (PPI) sub‐network based on significant (D) or group (E) analysis. Functionally grouped network with terms as nodes linked based on their kappa score level (≥0.3). The node size indicates significant enrichment.

Totally 286 differentially expressed genes (DEG) were found by RNA‐seq and differentially expressed genes analysis (*P‐*value < 0.05, fold change >2) of HS 737.T treated with paclitaxel. Among the 286 differentially expressed genes, upregulated and downregulated genes were 168 and 118, respectively (Fig. 2a); all these genes are shown in Supplementary Data 2 (upregulated genes) and 3 (downregulated genes).

As shown in Fig. 2b,c, the analysis revealed enrichment of functions correlated to the top 20 significantly canonical pathways of total DEG and upregulated genes, respectively. It also revealed that apoptosis, TNF signaling pathway, TP53 regulated transcription of cell death genes pathway, and longevity regulated pathway, which was the top 20 most enriched pathway and involved in cell viability (*P‐*value < 0.01).

A functional map of the upregulated gene protein–protein interactions (PPI) based on significant or group analysis revealed that apoptosis, TNF signaling pathway, TP53 regulated transcription of cell death genes pathway, and death receptor signaling pathway was the important regulatory node in paclitaxel‐driven apoptosis of HS 737.T cells, (the node size indicates significant enrichment), and the core components were related to apoptosis and viability of tumor cells, including TP53INPI, Caspase 10, TNFSF10, and PIK3R3 (Fig. 2d,e).

### *Certification by Real‐time Quantitative Polymerase Chain Reaction and Western Blotting*

**Figure 3:**
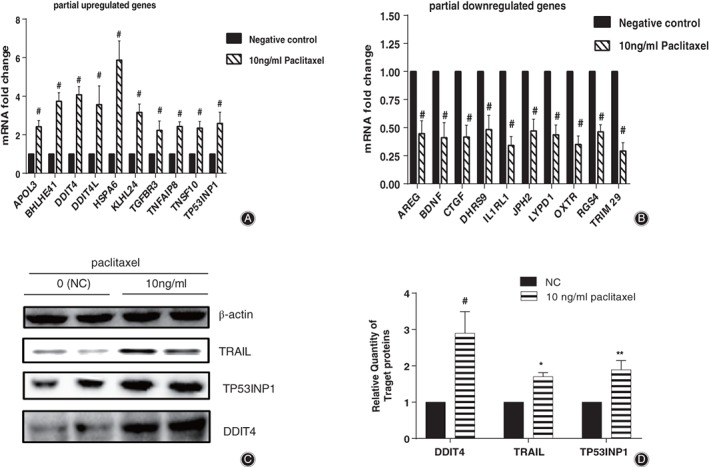
Validation of gene expression levels of partial differentially expressed genes. Verification of 10 upregulated (A) and downregulated (B) genes by quantitative real‐time polymerase chain reaction. (C) Western blot analysis validated 4 upregulated genes related with apoptosis. (D) Relative protein expression of TRAIL, TP53INP1, and DDIT4gene in (C). *P < 0.05, **P < 0.01, #*P* < 0.01 indicated statistical significance versus negative control.

Our results confirmed that the 10 upregulated and 10 downregulated genes which were associated with programmed cell death or cell longevity were significantly changed (*P‐*value < 0.001, fold change >2) in HS 737.T cells treated with paclitaxel, compared with the negative control, as analysis by RNA‐Seq demonstrated (Fig. 3a,b). These results showed that data for RNA‐seq and RT‐qPCR were highly consistent. In addition, western blot results showed that DDIT4, TP53INP1, and TRAIL were upregulated in HS 737.T cells compared with the negative control, and the increase was 183%, 87%, and 68%, respectively (Fig. 3c,d).

### *Increased Cleavage of Caspase‐3 and PARP1*

**Figure 4:**
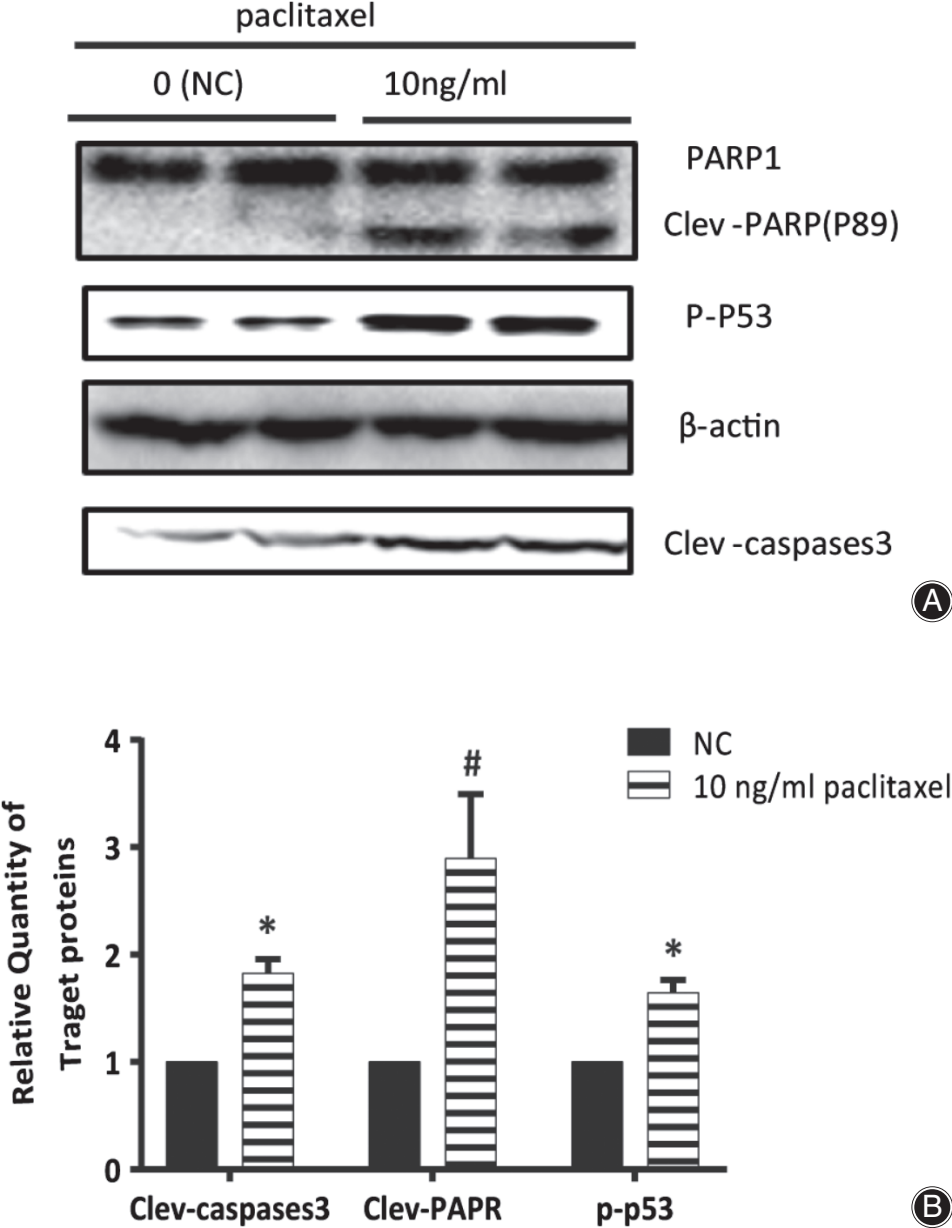
Paclitaxel enhanced the cleavage of Caspase‐3 and PARP1, and promoted the phosphorylation level of p53 in HS 737.T cells. (A) Analysis of the amount of cleaved Caspase‐3 or PARP1, and phosphorylation level of p53 by western blot. (B) Density of cleavage of Caspase‐3 or PARP1, and phosphorylation level of p53 normalized to that of β‐actin; data from (A).

As shown in Fig. 4, paclitaxel enhanced the cleavage of Caspase‐3 and PARP1 in HS 737.T cells, and the increase was 80% and 190%, respectively.

### *Promoted Phosphorylation Level of p53*

p53 responds to various stimulus through modulation of objective genes which are involved in DNA repair, apoptosis or cell cycle, and it can be activated *via* phosphorylation^15^. Paclitaxel increased the phosphorylation level of p53 to 165% in HS 737.T cells, which implied that p53 might be involved in the paclitaxel‐driven apoptosis of HS 737.T cells (Fig. 4a,b).

### *Regulation of Paclitaxel‐driven Apoptosis by TP53INP1*

**Figure 5:**
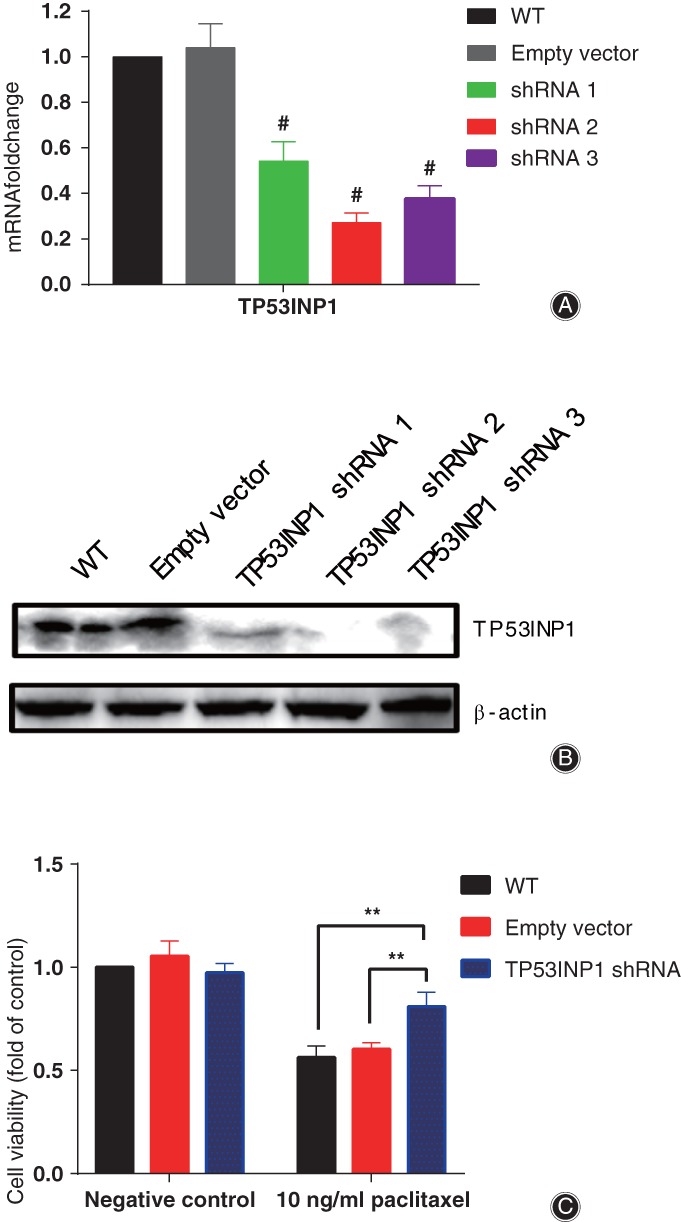
Tumor protein p53 inducible nuclear protein 1 (TP53INP1) regulated paclitaxel‐driven apoptosis in HS 737.T cells. RNAi efficiency of TP53INP1 in HS 737.T cells verified by quantitative real‐time polymerase chain reaction (A) and western blot (B). (C) Significant difference of cell viability among HS 737.T cells transfected with empty vector or TP53INP1‐shRNA by paclitaxel treatment. WT: wild type HS 737.T cell; empty vector: HS 737.T cells transfected with empty vector; TP53INP1‐shRNA: HS 737.T cells transfected with TP53INP1‐shRNA. *P < 0.05, **P < 0.01, #*P* < 0.01 indicated statistical significance versus negative control.

RNAi results showed that the expression level of TP53INP1 was significantly decreased in HS737.T cells; the decrease was more than 70% (Fig. 5a,b). We measured the viability of paclitaxel in TP53INP1‐knockdown cells. As expected, HS737.T cells deficient in TP53INP1 were insensitive to paclitaxel treatment compared with empty vector. The inhibitory ratios of HS737.T cells deficient in TP53INP1 or with empty vector were 19.88% and 40.6%, respectively (Fig. 5c). Therefore, these data illustrated that TP53INP1 regulated paclitaxel‐driven apoptosis in HS737.T cells.

## Discussion

Accumulating evidence has indicated that paclitaxel binds to b‐tubulin and increases the instability of microtubules, resulting in apoptosis in various cell lines^16^, ^17^. Repression of microtubule dynamics leads to mitotic arrest, and micronucleation and apoptosis arise in succession^18^.

In this study, paclitaxel inhibited the proliferation of HS 737.T cells in a dose‐dependent and time‐dependent manner (Fig. 1a,b). Further experiments showed that paclitaxel inhibited the proliferation of HS 737.T cells mainly *via* apoptosis (Fig. 1c,d).

For most anticancer agents, apoptosis is thought to be a crucial mechanism that represses the proliferation of cancer cells. To further investigate the mechanisms through which paclitaxel triggered the apoptosis of HS 737.T cells, we measured the differential expression of genes using RNA‐Seq and analyzed them by bioinformatics assay. Paclitaxel treatment upregulated the expression of genes involved in apoptosis, TP53 regulated transcription of cell death genes, and the TNF signaling pathway, including TRAIL, Caspase‐10, TP53INP1, and DDIT4.

Upon TRAIL binding to its receptor DR4/DR5, it speedily triggers apoptosis through caspases’ activation^19^. When binding with TRAIL, DR4/DR5 activates Caspase‐8^20^ and Caspase‐10. The activated caspases complex initiates Caspase‐3 and leads to the death of apoptotic cells. Our results demonstrated that paclitaxel treatment promoted the expression of TRAIL (TNFSF10) and Caspase‐10 (Figs 2 and 3), and activated Caspase‐3 (Fig. 4). Our data further demonstrated that paclitaxel‐driven apoptosis was triggered by TRAIL and its receptor DR4/DR5, and was dependent on Caspase‐8 and Caspase‐10 complex, which then resulted in the activation of downstream effector Caspase‐3.

Activated Caspase‐3 induces apoptosis *via* modulation of diverse target genes, such as DFF45, Rock1, and PARP1. However, which gene was involved in this process remained unknown. Herein, we determined the cleavage of Caspase‐3 and PARP1 in paclitaxel‐treated or untreated HS 737.T cells. As in Fig. 4, PARP1 cleavage was increased by treatment with 10 ng/mL paclitaxel. It has been demonstrated that PARP‐1 plays a vital role in DNA repair and various pathways of cell death^21^, ^22^. PARP1 is the substrate of Caspase‐3, and its cleavage has been widely used as a biochemical marker of apoptosis^23^. The cleavage of PARP1 was obviously raised, which suggested that paclitaxel‐driven apoptosis might be *via* repression of DNA repair.

p53 is a transactivating protein containing DNA‐binding and transcription activation domains, and contributes to apoptosis induction mostly through its transcription‐dependent effects. It is thought to bind to a p53‐binding site and to activate expression of downstream genes, which represses cell growth and promotes apoptosis; stabilized p53 accumulates in the nucleus and binds to specific DNA sequences, leading to transactivation of several pro‐apoptosis genes. Alternatively, cytoplasmic p53 can induce cell death *via* apoptosis and act as a repressor of autophagy^24^, ^25^, ^26^. In the present study, the phosphorylation level of p53 was significantly increased in HS 737.T cells treated with paclitaxel (Fig. 4a,b). These results suggested that paclitaxel‐driven apoptosis might be partially associated with P53.

Tumor protein p53 inducible nuclear protein 1 (TP53INP1) is an anti‐proliferative and pro‐apoptosis protein involved in cell stress response^27^, ^28^, ^29^, ^30^. In response to intracellular reactive oxygen species and double‐strand DNA breaks, TP53INP1 promotes p53 phosphorylation and triggers subsequent apoptosis in a caspase‐dependent pattern^31^, ^32^. The expression of TP53INP1 and the phosphorylation level of p53 were significantly increased in HS 737.T cells treated with paclitaxel (Fig. 3a,c,d). These results implied that paclitaxel‐activated TP53INP1 in the GCTB cells was involved in triggering apoptosis and cell death. Therefore, we determined the impact of paclitaxel on the viability of TP53INP1‐deficient HS 737 T cells. Interestingly, TP53INP1‐deficient HS 737.T cells were relatively insensitive to paclitaxel treatment (Fig. 5c), which signaled that knockdown TP53INP1 blocked paclitaxel‐induced cell death and apoptosis. The RNAi‐based inhibitory experiments revealed that TP53INP1 was crucial in HS 737.T cell apoptosis driven by paclitaxel.

**Figure 6:**
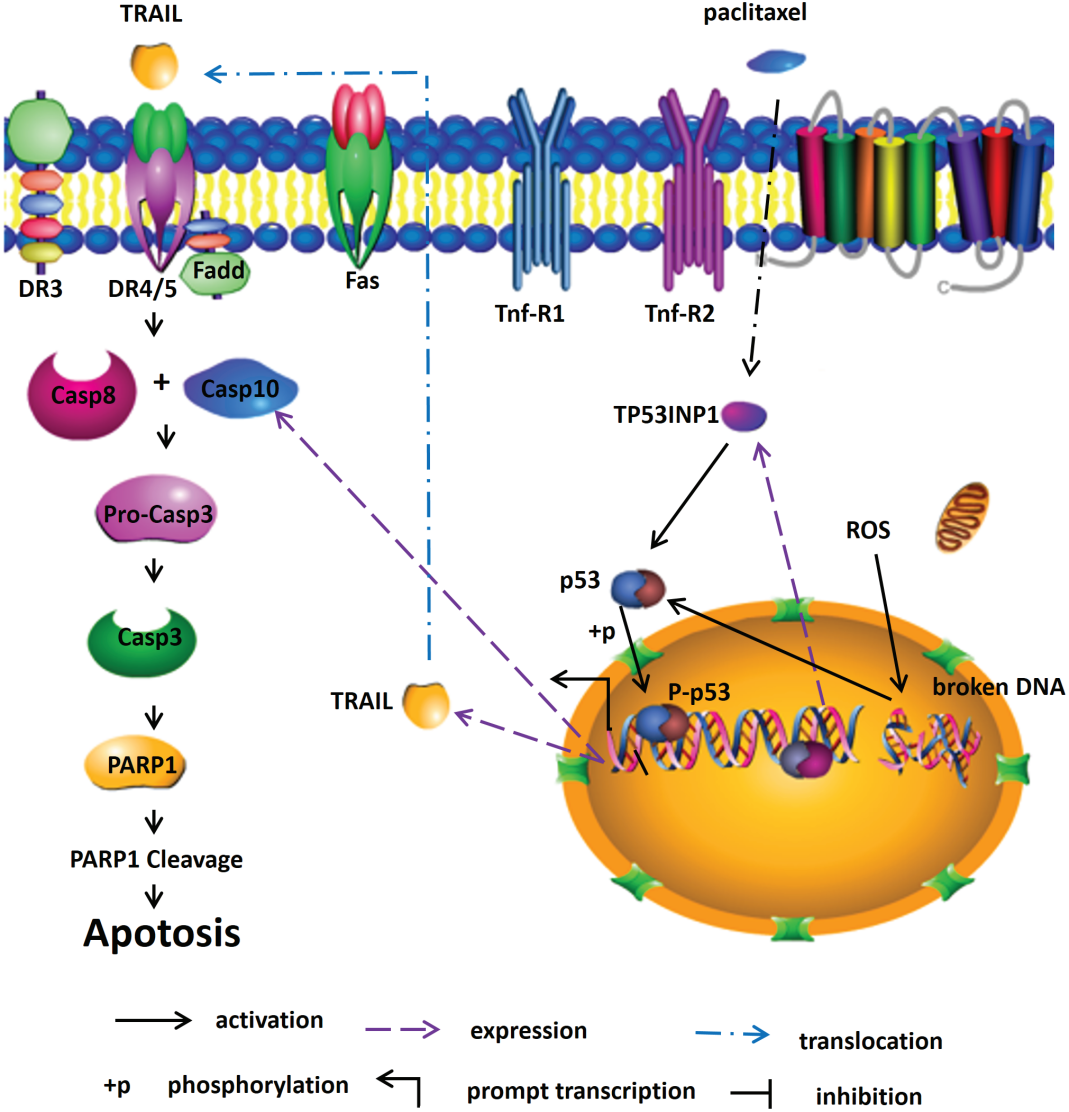
Schematic of paclitaxel driven‐apoptosis in HS 737.T cells through TP53INP1 signaling pathways.

As vital anti‐tumor drugs, most chemotherapeutic agents rely on the capability to induce apoptosis of tumor cells. To further develop paclitaxel as an effective anti‐GCTB agent in the clinic, we need to better comprehend the mechanisms through which paclitaxel drives apoptosis of GCTB. Based on the results of the present study, a model of the mechanism through which paclitaxel triggers apoptosis of HS 737.T cells was proposed (Fig. 6). Our data signaled that paclitaxel activated TP53INP1 and p53, which increased the expression of TRAIL, and subsequently induced apoptosis of GCTB cells.

There are some limitations of this study. First, we studied the apoptosis mechanisms of paclitaxel based on only one kind of GCTB cell line. Further studies could use more cell lines to investigate the commonness and mechanisms of paclitaxel‐driven apoptosis of GCTB. Second, we lacked data from animal experiments. Future research should consider experiments on transplanted tumors in animals.

In summary, by combining transcriptomics, bioinformatics, and RNAi (Lentivirus shRNA), we revealed systemic transcriptomics changes in HS 737.T cells under paclitaxel treatment and the therapeutic mechanism of the treatment. Results of RNA‐seq and bioinformatics analysis showed that apoptosis, death receptor signaling pathway, TNF signaling pathway, and TP53 regulated transcription of cell death genes pathway, which was the top 20 most enriched pathway and involved in cell viability, were closely associated with paclitaxel in the treatment of GCTB. Combined with transcriptomics and RNAi, TP53INP1 signaling pathways were ultimately found to be closely related to paclitaxel in the treatment of GCTB. Our study implied that paclitaxel might be valuable for the repression of GCTB.

## Supporting information


**Table S1** Primers specific for quantitative real‐time polymerase chain reaction
**Table S2** shRNA primers for TP53INP1
