# Supplementary material for: Paclitaxel Induce Apoptosis of Giant Cells Tumor of Bone via TP53INP1 Signaling
Source: Orthop Surg. 2018 Dec 27;11(1):126–34. doi: 10.1111/os.12414 (PMC6430463; doi:10.1111/os.12414)
Supplement: Supplementary file 1 — Table S1 Primers specific for quantitative real‐time polymerase chain reaction Table S2 shRNA primers for TP53INP1 [file OS-11-126-s001.zip › os12414-sup-0001-AppendixS1.docx]

Supplementary Table 1 Primers specific for RT-qPCR

| **Gene** | **Forward primer** | **Reverse primer** |
| --- | --- | --- |
| APOL3 | CCAGCATTGACCGATTGAAGG | TGATGGCACGGATTTCACTCC |
| BHLHE41 | AAGGAGCATGAAACGAGACGA | CTCGGTTAAGGCGGTTAAAGC |
| DDIT4 | TGAGGATGAACACTTGTGTGC | CCAACTGGCTAGGCATCAGC |
| DDIT4L | TGTGGCTATCACCCAGAGAG | GCTTTGATTTGGACAGACAGT |
| HSPA6 | GATGTGTCGGTTCTCTCCATTG | CTTCCATGAAGTGGTTCACGA |
| KLHL24 | ATATTGGGACGCAGACTAAACAG | TGTCAGAGATTTGGGGTCCAT |
| TGFBR3 | GTGTTCCCTCCAAAGTGCAAC | AGCTCGATGATGTGTACTTCCT |
| TNFAIP8 | CTTTGACCGGAATGTGTTATCCA | CAAGGCAGCCAAAAATTCACAA |
| TNSF10 | TGCGTGCTGATCGTGATCTTC | GCTCGTTGGTAAAGTACACGTA |
| TP53INP1 | TTCCTCCAACCAAGAACCAGA | GCTCAGTAGGTGACTCTTCACT |
| AREG | GAGCCGACTATGACTACTCAGA | TCACTTTCCGTCTTGTTTTGGG |
| BDNF | GGCTTGACATCATTGGCTGAC | CATTGGGCCGAACTTTCTGGT |
| CTGF | AAAAGTGCATCCGTACTCCCA | CCGTCGGTACATACTCCACAG |
| DHRS9 | ACCTCAGAGAGACTTCGTACTG | AGTCCAAACAGGTTCACTTCAAT |
| IL1RL1 | AGAAATCGTGTGTTTGCCTCA | TCCAGTCCTATTGAATGTGGGA |
| JPH2 | ACTCTGGCTCCTGGAACTTTG | GCGCCCCTTGGTCTCTATG |
| LYPD1 | GGCAACTTTTTGCGGATTGTT | CGTTCACCGTGCAATTCACA |
| OXTR | CTGCTACGGCCTTATCAGCTT | CGCTCCACATCTGCACGAA |
| RGS4 | ACATCGGCTAGGTTTCCTGC | GTTGTGGGAAGAATTGTGTTCAC |
| TRIM29 | AGCATCAGCGACTCTGTGTTG | GAAGTTGCCTAGTGACTGTCC |

Supplementary Table 2 shRNA primers for TP53INP1

|  | **primer sequences (5’--3’)** |
| --- | --- |
| **Forward 1** | CCGGCATAGATACTTGCACTGGTTTCTCGAGAAACCAGTGCAAGTATCTATGTTTTTG |
| **Reverse 1** | AATTCAAAAACATAGATACTTGCACTGGTTTCTCGAGAAACCAGTGCAAGTATCTATG |
| **Forward 2** | CCGGGCGCCATGTTTCTCAAAGTTTCTCGAGAAACTTTGAGAAACATGGCGCTTTTTG |
| **Reverse 2** | AATTCAAAAAGCGCCATGTTTCTCAAAGTTTCTCGAGAAACTTTGAGAAACATGGCGC |
| **Forward 3** | CCGGCCATGCAAACTGTTCCTGTTTCTCGAGAAACAGGAACAGTTTGCATGGTTTTTG |
| **Reverse 3** | AATTCAAAAACCATGCAAACTGTTCCTGTTTCTCGAGAAACAGGAACAGTTTGCATGG |
